# Extended Rate Constants Distribution (RCD) Model for Sorption in Heterogeneous Systems: 4. Kinetics of Metal Ions Sorption in the Presence of Complexing Agents—Application to Cu(II) Sorption on Polyethyleneimine Cryogel from Acetate and Tartrate Solutions

**DOI:** 10.3390/ijms241512385

**Published:** 2023-08-03

**Authors:** Alexey Golikov, Yuliya Privar, Denis Balatskiy, Natalia Polyakova, Svetlana Bratskaya

**Affiliations:** Institute of Chemistry, Far Eastern Branch of the Russian Academy of Sciences, 159, Prospect 100-Letiya Vladivostoka, 690022 Vladivostok, Russia; glk@ich.dvo.ru (A.G.);

**Keywords:** polyethyleneimine, porous material, cryogel, sorption kinetics, sorption mechanism, FTIR spectroscopy

## Abstract

Here, we report a new version of the extended Rate Constants Distribution (RCD) model for metal ion sorption, which includes complex-formation equilibria. With the RCD-complex model, one can predict sorbent performance in the presence of complexing agents using data on metal ion sorption from ligand-free solutions and a set of coefficients for sorption rate constants of different ionic species. The RCD-complex model was applied to breakthrough curves of Cu(II) sorption from acetate and tartrate solutions on polyethyleneimine (PEI) monolith cryogel at different flow rates and ionic speciation. We have shown that, despite the lower stability of Cu(II)-acetate complex, at high flow rates, acetate has a more pronounced negative effect on sorption kinetics than tartrate. The RCD model was successfully used to predict the shape of the breakthrough curves at an arbitrary acetate concentration but failed to predict Cu(II) sorption from tartrate solutions in a broad range of ligand concentrations. Since a twofold increase in sorption capacity was observed at low tartrate concentrations, the latter fact was related to an alteration in the sorption mechanism of Cu(II)-ions, which depended on Cu(II) ionic speciation. The obtained results emphasize the importance of information about sorption kinetics of different ionic forms for the optimization of sorption filter performance in the presence of complexing agents.

## 1. Introduction

The problem of heavy metal removal from natural and waste water remains in focus, despite technological achievements and the availability of a broad selection of different types of sorbents [1,2,3,4,5,6]. Aside from affinity, selectivity, and high sorption capacity, the kinetic characteristics of sorbents are of utmost importance as they determine the productivity of both industrial water treatment facilities and small-size point-of-use (POU) filters. Demand for materials, which can efficiently remove metal ions at high flow rates in fixed-bed applications has resulted in a sharp growth in demand for highly porous sorbents that are based on water-soluble synthetic and natural polymers [4,5,6,7,8,9,10,11]. However, when diffusion limitations are eliminated due to the well-developed porous structure, chemisorption (surface chemical reaction) can become a rate-limiting stage of adsorption. In this case, even in single metal solutions, metal speciation can significantly affect not only equilibrium sorption parameters but also sorption kinetics via an alteration of the sorption mechanism and a difference in the sorption/desorption rate constants for different species. 

Ligands can alter both metal speciation and solid surface properties, leading to negative or positive cooperative effects on adsorption, depending on adsorption mechanisms, pH, nature of the metal ion, ligands and adsorbent, and their ratios. For example, the efficiency of Cr(VI) adsorption on polyethylenimine (PEI)/chitosan aerogel beads decreased significantly in the presence of PO_4_^3−^ anion, which strongly competed with HCrO_4_^−^ for the active sorption site [12]. At the same time, phosphates had a generally promotive effect on metal ion sorption on different minerals due to the combination of surface electrostatic effects, ternary surface complexation, and surface precipitation [13]. Pre-adsorption of tartrate on the mixed Fe-Al oxides was shown to promote Pb(II) binding due to ternary complex formation [14,15]. The removal of Cu(II) by Fe_3_O_4_/GO/DCTA was enhanced by the tartrate presence at pH < 6.5 due to the formation of hydrogen bonds between the Cu(II)-tartrate complex and carboxylic groups at the sorbent surface [15]. Sorption capacity of multi-amines decorated resin (PAMD) for Cu(II) was found to increase from 1.77 mmol/g to 5.07 mmol/g in the presence of citric acid, which was explained by the alteration of the mechanism from single-site to dual-site binding. In this dual-site binding, cationic or neutral Cu species (Cu^2+^ and CuHL^0^) were coordinated with neutral amine sites, and anionic complex species (CuL^−^ and Cu_2_L_2_^2−^) directly interacted with protonated amine sites via electrostatic attraction [16].

It is worth mentioning that the effect of complexing agents on the metal sorption kinetics is not well investigated. Environmental models focus on equilibrium metal ion speciation and mineral surface charge and neglect sorption kinetics [13]. Kinetics of metal ion removal from solutions containing metal complexes are most often investigated in batch [16,17] or at relatively low for porous materials flow rates (up to 25 BV/h) [12,16,18,19]. However, even under these conditions, it was demonstrated that the sorption rate constants for Pb(II) and Cu(II) species could differ for more than one log unit in the presence of citric acid and EDTA [16,17]. An unusual effect was observed for Cu(II) sorption on PEI-agarose adsorbent from 0.1 M acetate buffer, when the total copper uptake was doubled in the presence of citrate in fixed-bed applications at a flow rate of 25 BV/h, while in batch experiments binding capacity of PEI was the same regardless whether or not citric acid was present in the solution [18]. This finding contradicts the conclusion about significant increase in Cu(II) uptake on PAMD as reported in a previous study [16].

These examples demonstrate that knowledge on sorption kinetics of different ionic species is crucial to optimize and predict performance of sorption materials in practice, especially for the filters designed for applications at high flow rates in solutions containing complexing agents. Different sorption kinetic models are used to reveal the rate-limiting mechanism and extrapolate the kinetic parameters for the operating conditions of interest [20]. Many simplified phenomenological models that assume diffusion- [21] or reaction-controlled [22] sorption kinetics, which are widely used to fit experimental kinetic curves with a limited number of adjustable parameters, often lack physical meaning [20,23]. Kinetic curves can also be considered as a sum of fragments with different limiting factors, so a separate kinetic model is applied for each fragment [20,24,25]. However, in most cases, for each sorption stage, the sorption rate is affected by several factors, which are difficult to separate [20,25,26]. Moreover, sorbent surface heterogeneity can complicate such artificial fragmentation of the kinetic curve. As a result, the predictive potential of most kinetic models is rather low, so numerous time- and cost-consuming tests are required to predict shapes of the breakthrough curves under different sorption conditions and sorption column geometries. 

To overcome the limitations of conventional kinetic models, we have recently developed and verified the extended Rate Constants Distribution (RCD) model [27,28,29], which is based on the Langmuir kinetics model [30,31,32,33,34], linking a well-developed theoretical description of the adsorption equilibrium and pseudo-first- (PFO) or pseudo-second-order (PSO) rate kinetics equations. Since the chemical equilibrium is a balance between forward (adsorption) and reverse (desorption) rates of reaction, the equilibrium and kinetic constants can be determined through complete kinetic measurements [30]. However, due to the mathematical and computational challenges, the extended RCD model [27,28,29] became the first in which both adsorption and desorption rate constants were determined for heterogeneous sorbents via simultaneous processing of several experimental curves of sorption kinetics in batch or in fixed-bed applications. This single RCD function, which describes the fill set of experimental curves, contains all information about affinity, quantity, and distribution of the sorption sites in the space of constants of sorption and desorption rates for heterogeneous sorbent and can be used to predict distribution of the adsorbate on sorption centers for different starting conditions (solid:liquid ratio or column geometry, adsorbate concentration, and flow rate) at any time of the sorption process. 

Recently, we reported the fabrication of polyethylenimine (PEI) monolith supermacroporous cryogels with high efficiency of heavy metal ions under dynamic conditions at flow rates up to 160 bed volume (BV)/h [35]. Application of the RCD model to Cu(II), Ni(II), Zn(II), and Cd(II) sorption on PEI cryogel in batch and fixed-bed experiments [27,29] allowed identification of “fast” and “slow” sorption centers. This model also provided an explanation for the preferential adsorption of one or another ion from the mixture in a fixed-bed application, depending on the experimental conditions such as flow rate and metal ion concentrations [29]. Moreover, RCD functions for Cu(II), Zn(II), and Cd(II) sorption on PEI cryogels obtained from the batch data were successfully used for the first time to predict the rate dependence of a breakthrough point position and shape of the breakthrough curves in a fixed-bed application for a broad range of adsorbate concentrations and flow rates [29]. 

However, in the presence of complexing agents, metal ion speciation in solutions differs depending on metal/ligand ratio and pH; thus, RCD functions obtained for metal cations sorption in ligand-free solutions cannot be used to model sorption kinetics in the presence of ligands. Obviously, another RCD function can be obtained for the sorption of a metal complex, but its application will be limited to the systems containing only one ionic form of the metal and will not be applicable to the broad range of metal/ligand ratios and mixture of ionic species. This problem can be resolved if we extend the RCD model with complex-formation equilibria and find constants of sorption/desorption rates for all possible ionic forms. Mathematically, it can be more easily performed for the systems for which binding constants of a metal ion to the sorbent functional groups will significantly exceed the binding constant to a ligand. In other words, the structure of the metal–sorbent complex will be the same after the sorption from ligand-free solution and solutions containing different metal–ligand species. So, if one can somehow modify RCD function for the metal ion sorption from ligand-free solution to take into account complex-formation equilibria, one will be able to perform predictive modeling of the breakthrough curves of metal sorption at various flow rates and metal/ligand ratios. 

Such systems, containing ligands, which form relatively weak complexes with transition metal ions, are common in industry and in the environment. One example is low-molecular-weight carboxylic acids, which are extensively used for soil remediation, leaching of metal ores [36], electroplating [37], and circuit-board printing, and thus shall be considered as co-contaminants in metal ion removal by sorption methods. The effect of their presence on the kinetics of metal ion removal in a fixed-bed application at high flow rates is important for wastewater treatment and an estimation of the sorption filters’ productivity.

Thus, in this work, which continues a series of papers on RCD model development and applications [27,28,29], we investigated how the presence of acetates and tartrates affects Cu(II) sorption in a fixed-bed application on a monolith supermacroporous PEI cryogel at different flow rates and metal/ligand ratios. Moreover, we verified whether the possibility of the RCD model extension to predict kinetics of the metal ion sorption in the presence of complexing agents is dependent on the sorption mechanism. 

## 2. Results and Discussion

### 2.1. Brief Introduction to RCD Model

The Extended Rate Constants Distribution (RCD) model for sorption in heterogeneous systems belongs to the pool of models, which assumes the presence of a continuum of different sorption sites [38,39,40,41]. The background and advantages of this model were verified and discussed in our earlier works [27,28,29]. The following assumptions were made in the RCD model: (1) the flow of adsorbate from the bulk to the sorbent is proportional to the adsorbate concentration in the solution and to the surface area with vacant sorption sites; (2) the flow of adsorbate from the surface to the bulk is proportional to the surface area with occupied sorption sites; and (3) the specific surface area occupied with sorption sites of the certain type is proportional to the content of such sites in the sorbent. 

The differential mass balance equation for a fixed-bed column is given in [42]:

(1) $$u\frac{\partial C}{\partial z}+\frac{\partial C}{\partial \tau }+\frac{1-\varepsilon }{\varepsilon }\rho_{p}\frac{\partial Q}{\partial \tau }=D_{L}\frac{\partial^{2}C}{\partial z^{2}}$$

where u is the superficial velocity, C is the adsorbate concentration in the solution, *z* is the axial coordinate, *τ* is the time, *ε* is the column void fraction, ρ is the adsorbent density, *Q* is the adsorbate content in the sorbent, and *D_L_* is the axial dispersion coefficient, which was set here to zero as in most other works on sorption dynamics [42,43]. 

Any suitable rate expression for *∂Q/∂τ* can be used to complete the fixed-bed model. Because, in most cases, metal ion adsorption is well described by Langmuir equation, in the RCD model we have used Langmuir adsorption kinetic Equation (2):

(2) $$\frac{dQ_{t}}{dt}=k_{s}C(Q^{\max}-Q_{t})-k_{d}Q_{t}$$

where *k_s_* is the second-order sorption rate constant, *k_d_* is the first-order desorption rate constant, *C*—the adsorbate concentration, *Q*^max^ is the total sorption capacity, and *Q*(*τ*) is the total content of the adsorbate in the sorbent at time *τ*.

To describe kinetics of sorption in the RCD model, we have introduced the density function *q*(*k_s_*, *k_d_*, *τ*), which shows the distribution of the adsorbate on sorption sites in the space of the rate constants (RC) of sorption (*k_s_*) and desorption (*k_d_*) at any time point (*τ*), and the density function *q*^max^ (*k_s_*, *k_d_*), which shows the maximal content of the adsorbate (sorption capacity) for the certain type of sorption sites (*k_s_, k_d_*) at full saturation. *Q*^max^ is the total sorption capacity. Using these functions, Equation (2) will transform to Equation (3):

(3) $$\frac{dq\left(k_{s},k_{d},\tau \right)}{d\tau }=k_{s}C\left(\tau \right)\left(q^{\max}\left(k_{s},k_{d}\right)-q\left(k_{s},k_{d},\tau \right)\right)-k_{d}q\left(k_{s},k_{d},\tau \right)$$

After addition of the material balance, Equation (4):

(4) $$Q^{0}+V_{sp}\cdot C^{0}=Q\left(\tau \right)+V_{sp}\cdot C\left(\tau \right)$$

where *Q*^0^ is adsorbate content in the sorbent, *C*^0^ is the adsorbate concentration in the solution in the initial time point, and *V_sp_* is the specific solution volume. For the heterogeneous sorbents, we can write the following integral equations, where Equation (6) describes the Langmuir-type sorption isotherm on a heterogeneous sorbent:

(5) $$\begin{array}{l} \int_{0}^{+\infty }\int_{0}^{+\infty }q\left(k_{s},k_{d},\tau \right)dk_{s}dk_{d}=Q\left(\tau \right)\\ \int_{0}^{+\infty }\int_{0}^{+\infty }q^{0}\left(k_{s},k_{d},0\right)dk_{s}dk_{d}=Q^{0} \end{array}$$

(6) $$Q^{e}=\int_{0}^{+\infty }\int_{0}^{+\infty }q^{\max}\left(k_{s},k_{d}\right)\frac{C^{e}k_{s}}{C^{e}k_{s}+k_{d}}dk_{s}dk_{d}$$

where *C^e^* and *Q^e^* are the equilibrium concentration of the adsorbate in the solution and the content of the adsorbate in the sorbent, respectively. Transformation of the systems of integro-differential Equations (3)–(6) to the form suitable for numerical calculations and processing the experimental data are described in [27]. 

### 2.2. RCD Model for Sorption in the Presence of Complexing Agents (RCD-Complex Model) 

Let us consider metal ion (Me) sorption on heterogeneous sorbent in the presence of complex-forming ligands (L). Assuming that n—maximal coordination number of metal ions, the following metal ionic forms will be present in solution in addition to the free metal cations: $MeL,MeL_{2},\ldots ,MeL_{n}$. The following equilibria can be written without taking into account ions charge (this is valid for the case when L—is not hydroxyl ion):

(7) $$\begin{array}{l} MeL_{n}\overset{k_{1}}{⇄}MeL_{n-1}+L\\ \ldots \\ MeL\overset{k_{n}}{⇄}Me+L \end{array}$$

$k_{1}\ldots k_{n}—$the stepwise complex instability constants. Material balance equation for metal is:

(8) $$N_{Me}^{0}=v\left(C_{Me}+\sum_{i=1}^{n}C_{MeL_{i}}\right)+\sum_{i=0}^{m-1}q_{i}$$

where v—the solution volume per mass unit of the sorbent; $q_{i}—$the content of metal on the i—the sorption center; m—the number of sorption centers; and $N_{Me}^{0}—$the initial number of moles of metal in solution in sorbent. The equation of material balance with respect to the ligand is:

(9) $$N_{L}^{0}=v\left(C_{L}+\sum_{i=1}^{n}i\cdot C_{MeL_{i}}\right)$$

$N_{L}^{0}—$the initial number of moles of a ligand; $C_{L}—$the current concentration of free ligands. 

If we assume momentary establishment of equilibrium concentrations of ionic forms in the solution, then to calculate equilibrium concentrations of ionic forms in the initial solution (without the sorbent), the equations of the material balance must be supplemented with the law of acting masses for all ionic forms:

(10) $$k_{i}C_{MeL_{n+1-i}}-C_{MeL_{n-i}}C_{L}=0,\;i=1\ldots n$$

Thus, we obtain the following system of nonlinear equations:

(11) $$\begin{array}{l} C_{Me}^{0}-C_{Me}-\sum_{i=1}^{n}C_{MeL_{i}}=0\\ C_{L}^{0}-C_{L}-\sum_{i=1}^{n}i\cdot C_{MeL_{i}}=0\\ k_{i}C_{MeL_{n+1-i}}-C_{MeL_{n-i}}C_{L}=0,\;i=1\ldots n \end{array}$$

Equation (11) was solved by the Newton–Raphson method to ensure the positive sign of concentrations of all ionic forms. Through solving Equation (11), one obtains the distribution in the forms of existence of metal ions for the initial time point. At the other time points, Equation (11) in differential forms was included in the system of differential equations of sorption kinetics and considered during integration:

(12) $$\begin{array}{l} -\frac{dC_{Me}\left(\tau \right)}{d\tau }-\sum_{i=1}^{n}\frac{dC_{MeL_{i}}\left(\tau \right)}{d\tau }-\frac{1}{v}\sum_{i=0}^{m-1}\frac{dq_{Me}\left(k_{s,Me},k_{d,Me}\right)}{d\tau }=0\\ -\frac{dC_{L}\left(\tau \right)}{d\tau }-\sum_{i=1}^{n}i\cdot \frac{dC_{MeL_{i}}\left(\tau \right)}{d\tau }=0\\ k_{i}\frac{dC_{MeL_{n+1-i}}\left(\tau \right)}{d\tau }-\frac{dC_{MeL_{n-i}}\left(\tau \right)}{d\tau }C_{L}\left(\tau \right)-C_{MeL_{n-i}}\left(\tau \right)\frac{dC_{L}\left(\tau \right)}{d\tau }=0,\;i=1\ldots n \end{array}$$

Let us assume that (1) the final form of binding of any complex metal forms with the sorbent surface—Me; (2) all the complex ionic forms Me are sorbed (with different desorption rates until 0); and (3) the constants of sorption rates for metal complex forms are related through functional dependencies with sorption/desorption rates of the ion Me (if we know the function of distribution of metal ions without the complex ion). Therefore, the sorption equation (just like Equation (3) in Section 2.1 of the Langmuir sorption kinetics in the absence of the complexing agent) can be written as:

(13) $$\begin{array}{l} \frac{dq_{Me}\left(k_{s,Me},k_{d,Me}\right)}{d\tau }=\left(k_{s,Me}C_{Me}\left(\tau \right)+\sum_{i=1}^{n}K_{s,MeL_{i}}\left(pk_{s,Me},pk_{d,Me}\right)C_{MeL_{i}}\left(\tau \right)\right)\\ \times \left(q_{Me}^{\max}\left(k_{s,Me},k_{d,Me}\right)-q_{Me}\left(k_{s,Me},k_{d,Me}\right)\right)-k_{d,Me}q_{Me}\left(k_{s,Me},k_{d,Me}\right)\\ pk=\ln\left(k\right) \end{array}$$

where $K_{s,MeL_{i}}\left(pk_{s,Me},pk_{d,Me}\right)—$the function of dependence of the rate of sorption of the ionic form $MeL_{i}$ on logarithms of constants of sorption/desorption rates of the ion Me; $q_{Me}\left(k_{s,Me},k_{d,Me}\right)—$the function of distribution of the content of the ion Me over sorption centers; and $C_{Me}\left(\tau \right),C_{MeL_{i}}\left(\tau \right)—$the concentration of ion and ionic forms of Me in solution in the time τ. The appearance of the functions $K_{s,MeL_{i}}\left(pk_{s,Me},pk_{d,Me}\right)$ is an unknown *a priori*, but they can be expanded into a Taylor series around the point $\left(pk_{s,Me},pk_{d,Me}\right)$:

(14) $$K_{s}\left(pk_{s},pk_{d}\right)=a_{00}+a_{10}pk_{s}+a_{01}pk_{d}+a_{20}pk_{s}^{2}+a_{11}pk_{s}pk_{d}+a_{02}pk_{d}^{2}+\ldots$$

where $a_{ij}—$the coefficient of a two-dimensional Taylor series by the term $k_{s}^{i}k_{d}^{j}$ (see Appendix A for details).

To sum up, if one knows the function of distribution of sorption centers for the ion Me, then to describe sorption of metal complex forms, one must determine the coefficients $a_{ij}$ for every complex form. The latter can be easily realized through minimization of the functional below (taking into account the solution regularization):

(15) $$\begin{array}{l} \underset{a_{MeL},a_{MeL_{2}},\ldots }{\min}\;F\left(a_{MeL},a_{MeL_{2}},\ldots \right)=\sum_{i=1}^{M}\sum_{j=1}^{N_{i}}{\left(C_{ij,calc}\left(a_{MeL},a_{MeL_{2}},\ldots \right)-C_{ij,\exp}\right)}^{2}\\ +\lambda \Omega \left(a_{MeL},a_{MeL_{2}},\ldots \right)\\ \Omega \left(a_{MeL},a_{MeL_{2}},\ldots \right)=\sum_{k=1}^{n_{L}}\left\|a_{MeL_{k}}-a_{0,MeL_{k}}\right\|\;\\ a_{0,MeL_{k}}=\left(0,1,0\ldots 0\right) \end{array}$$

where $a_{MeL_{i}}—$the vector of unknown coefficients in the expansion (14) for the ionic form $MeL_{i}$; $M,N_{i}—$the number of experimental kinetic curves for sorption of complex forms of Me and the number of experimental points on the curve i; $C_{ij,calc}\left(a_{MeL},a_{MeL_{2}},\ldots \right)—$the calculated value of the metal ion concentration in the point i on the curve j for specific values of the vector components $a_{ij}$; $\Omega \left(a_{MeL},a_{MeL_{2}},\ldots \right)—$the solution stabilizer; λ—the regularization parameter; $\left\|a_{MeL_{k}}-a_{0,MeL_{k}}\right\|—$the Euclid form of the difference between the “zero” and current vector $a_{MeL_{k}}$ (i.e., it is preferable that the optimal vector of expansion coefficients would not differ significantly from the “zero” one); and $n_{L}—$the maximal degree of complex binding of the metal ion. 

To sum up, in order to describe the metal sorption in the presence of the complexing agent, it is first necessary (i) to determine the function of density of sorption centers for sorption of pure Me; (ii) to obtain the number of sorption kinetic curves from metal–chelate solutions, preferably, differing in metal–ligand concentrations; (iii) to find an adequate set of coefficients in expansion (14) via the functional minimization (15). The initial values of the vector $a_{ij}$ components are determined by the conventional method (the constants of rates for all the complex forms are equal to those of metal ions):

(16) $$a_{10}=1.0;\;a_{ij}=0.0,\;ij\neq 10$$

In other words, the initial values of constants of rated for all the ionic forms are equal to those of metal ions.

### 2.3. Cu(II) Soprtion on PEI Cryogel in the Presence of Acetate and Tartrate 

Fabrication and characterization of PEI cryogels using diglycidyl ether of 1,4-butanediol (DGEBD) as a cross-linker was reported earlier in [35]. At a molar ratio DGEBD: PEI of 1:4, we obtained a highly permeable monolith cryogel with a swelling degree of 2200% and a pore size of 128 ± 30 μm, which supported a liquid flow rate up to 450 BV/h [35]. Efficient mass transfer through the channels of interconnected macropores under dynamic conditions and very low thickness (6.1 ± 2.6 μm) of the polymeric walls assured very high rates of metal ion sorption in this cryogel via coordination mechanism [29]. However, in case of electrostatic interactions between positively charged PEI and anionic adsorbates (alizarin red dye and humic acid), an increase in the surface coverage reduced attractive forces between the adsorbate, so the sorption rate slowed down and breakthrough curves featured an asymmetric shape with a bend after the breakpoint and significant difference between effective dynamic sorption capacities at moderate flow rates. 

To investigate how the presence of acetic and tartaric acids as complexing agents affect ability of PEI cryogel to remove Cu(II) ions under dynamic conditions, we have designed several model solutions with different compositions of metal ionic forms using Phreeqc Interactive 3.7.3-15968 software (Table 1 and Table 2). Taking into account that wastewaters can contain a much higher excess of the complexing agents than were earlier used for studying Cu(II) sorption on PEI and polyamine resin in the presence of citric acid and EDTA (metal:ligand 1:2) [16,18,19], acetate and tartrate concentrations were varied over a broad range. 

**Figure 1:**
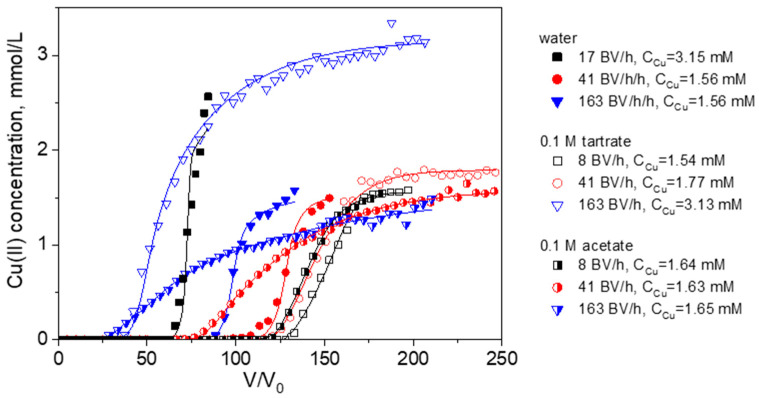
Breakthrough curves of Cu(II) ion sorption on PEI cryogel monoliths in a fixed-bed model from water and 0.1 M solutions of acetate and tartrate: dots are experimental data; lines are fits using the RCD model. Column parameters: diameter is 0.47 cm, bed length is 6 cm, sorbent weight is 0.085 g. Sorption conditions: pH = 5.2, T = 23 °C, flow rates (BV/h), and initial Cu(II) concentrations are as shown in the legend.

First, breakthrough curves of Cu(II) sorption from 0.1 M acetate and tartrate solutions were obtained at several flow rates from 8 to 163 BV/h and compared with those for Cu(II) sorption from water (Figure 1). Since the intraparticle diffusion in monolith PEI cryogel is not the rate-limiting stage of sorption [29,35], the difference in the breakthrough curve shape (slop and breakthrough point position) in water and ligand-containing solutions will reflect the difference in sorption rate constants of Cu(II) ionic species and a possible alteration of sorption mechanism.

Both acetate and tartrate Cu(II) complexes are significantly weaker in comparison with Cu(II)-citrate and Cu(II)-EDTA complexes (cumulative stability constant 14.2 and 18.7, respectively [44]). So, one can expect that, at equilibrium, Cu(II) ions will be bound by PEI cryogel with the release of the ligands to the solution. Considering the higher stability of Cu(II) tartrate complexes, tartrate could have a more profound effect on Cu(II) sorption than acetate. However, the effective dynamic sorption capacities for Cu(II) in 0.1 M tartrate were similar or even higher than those obtained in water, while the efficiency of Cu(II) uptake from 0.1 M acetate solution significantly dropped with an increasing flow rate (Figure 1, Table 3). The effective dynamic capacities were calculated for the breakthrough point of 2 mg/L that corresponds to the World Health Organization guideline value for copper in drinking water [45]. 

At flow rates of 41 BV/h and 163 BV/h, the effective sorption capacities of PEI for Cu(II) from 0.1 M acetate were only 80% and 50% of those in water, respectively. This shows that Cu(II) speciation in 0.1 M acetate solution significantly affects sorption kinetics despite the low thermodynamic stability of Cu(II)-acetate complex. From a practical point, this means that the presence of acetate will decrease filter productivity if the target of sorption process is to meet requirements for Cu content in discharge waters. 

**Figure 2:**
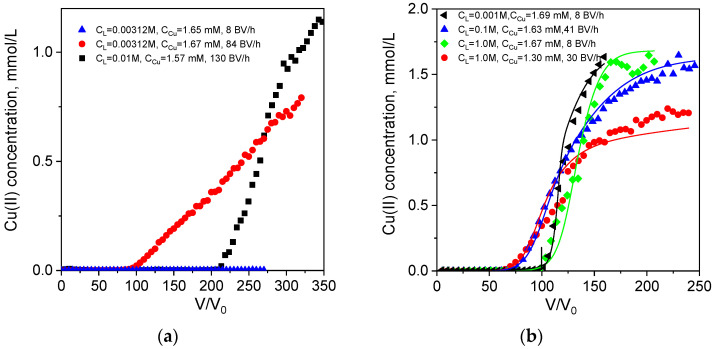
Breakthrough curves of Cu(II) ion sorption on PEI cryogel monoliths in a fixed-bed model from tartrate (**a**) and acetate (**b**) solutions: dots are experimental data; lines are fits using the RCD model. Column parameters: diameter is 0.47 cm, bed length is 6 cm, sorbent weight is 0.085 g. Sorption conditions: pH = 5.2, T = 23 °C, flow rates (BV/h), initial Cu(II) and ligand (L) concentrations are as shown in the legend.

In 0.1 M tartrate solution, Cu(II) exists predominantly in anionic form [CuL_4_]^6−^ (Table 2), which can be adsorbed by PEI via strong electrostatic interactions without a ligand-exchange reaction and the release of tartrate to the solution. This is a fast process and at the highest flow rate of 163 BV/h, effective dynamic sorption capacity for Cu(II) in tartrate is only 22% lower than that in water (Table 3). When tartrate concentration was decreased to a Cu(II):L ratio of 1:2, Cu(II) ionic speciation shifted toward a higher contribution (41.9%) of neutral form (0.00312 M tartrate, Table 2), and the increase in flow rate had a more profound effect of effective dynamic sorption capacity, which was threefold lower at 84 BV/h than at 8 BV/h (Figure 2a, Table 3). This allows us to identify neutral Cu(II) complex as a form with the lowest sorption rate. Indeed, when we increased tartrate concentration to 0.01 M, and contribution of all Cu(II) anionic forms increased from 11.3 to 43.4% (Table 2), effective dynamic sorption capacity was twice as high as in 0.00312 M tartrate (3.87 over 1.73 mmol/g), even at a higher flow rate of 130 BV/h (Table 3). These data are not in agreement with conclusion made for Cu(II) sorption from citrate solutions on polyaminated resin about the higher sorption rate constant for the neutral CuHL^0^ complex over anionic forms [16]. Regretfully, experimental data in [16] were obtained in batch and were not verified in fixed-bed models at different flow rates and metal/ligand ratios that could prove one or another hypothesis. 

At the same time, and similarly to the works [16,18,19,46] on Cu(II) sorption on polyamine resins in the presence of citrate, we observed a drastic increase in maximal sorption capacity for Cu(II) in the presence of a low excess of tartrate (Table 3). The maximal value of 5.61 mmol/g is in very good agreement with the sorption capacity of 5.5 mmol/g toward anionic dye earlier determined for this cryogel [35]. It should be mentioned that homogeneous distribution of Cu(II) in monolith cryogel after the sorption from both acetate and tartrate solutions was confirmed by SEM (Figure S1, Supplementary information). Thus, the difference in sorption capacities and rate-dependence of the shape of the breakthrough curves can be attributed to the different mechanisms of Cu(II) sorption in the presence of acetate and tartrate. 

### 2.4. Comments on Cu(II) Sorption Mechanism in Relation to Application of RCD-Complex Model 

**Figure 3:**
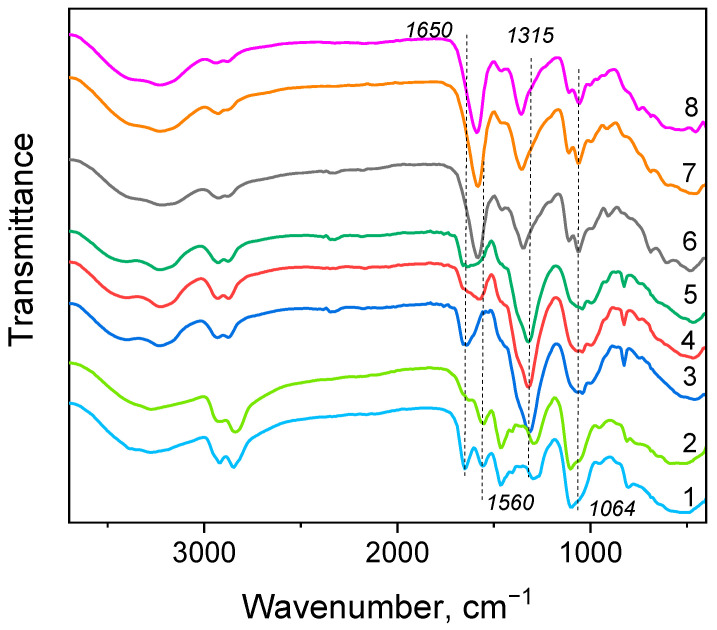
FT-IR spectra of PEI cryogels before (1, 2) and after (3–8) Cu(II) sorption: 1—PEI in H^+^ form; 2—PEI in OH^−^ form; PEI after Cu(II) sorption from water at 41 BV/h (3), from 0.1 M acetate at 41 BV/h (4), from 1 M acetate at 8 BV/h (5), from 0.1 M tartrate, 41 BV/h (6), from 0.01 M tartrate, 130 BV/h (7), from 3.12 mM tartrate, 8 BV/h (8). Dotted lines are given to guide the eyes.

There are numerous examples in the literature of when sorption mechanism changes depending on the ionic form of the adsorbate, even at the same pH value. Acetate buffers with high concentrations are often used as a background media to investigate metal sorption properties, since it is believed that acetate does not affect metal binding due to the low stability of acetate complexes [18]. Although we have demonstrated above that acetate can have profound effect on Cu(II) sorption kinetics, a comparison of FT-IR spectra (Figure 3) of PEI cryogels after Cu(II) sorption from water and acetate solutions reveals the following similar features: band shifts of N-H bond bending (1300–1315 and 1560 cm^−1^) and C-N bond stretching (1100–1050 cm^−1^) Cu(II) chelation by a different type of PEI amino groups. The notable difference was observed only in the region around 1650 cm^−1^, where bands of N-H bending can overlap with stretching vibration of carboxylic group. The widening and asymmetry of this band in PEI FT-IR spectra after Cu(II) sorption from both 0.1 M and 1 M acetate solutions does not exclude possibility of replacement of one water molecule with acetate in the coordination sphere of the PEI-Cu(II) complex, where copper has coordination number 5 [47]. However, it is important to emphasize that FT-IR spectra of PEI after the Cu(II) sorption in presence of acetate have the same features regardless of sorption conditions (ligand concentration and flow rate). 

In contrast, FT-IR spectra of PEI after Cu(II) sorption from 0.1 M, 0.01 M, and 3.12 mM tartrate solutions (spectra 6–8, Figure 3) feature significant changes in relative intensities and positions of the bands at 1315–1345 cm^−1^ and 1580–1650 cm^−1^ regions that can be interpreted in terms of overlapping N-H bending with asymmetric and symmetric stretching of carboxyl groups. Moreover, the shape of the peaks and area ratios depend on sorption conditions (Figure S2, Supplementary materials) that can reflect changes in the binding mode depending on ionic speciation [48]. Cu(II) sorption from tartrate solutions can be additionally complicated by formation of polynuclear complexes. Calculations of Cu(II) ionic speciation in 3 mM tartrate solution with a tartrate:Cu(II) molar ratio of 2:1: using stability constants for bi-nuclear tartrate complexes [49] showed that 7.45% of Cu(II) exists in the [Cu_2_L_2_]^0^ form (Table S3, Supplementary materials). Formation of such complexes can contribute to the increase in sorption capacity in solutions with low tartrate concentrations. However, Ling et al. [16] suggested that neutral Cu(II)-citrate complex was adsorbed on polyaminated resin with the release of the ligand via coordination with amine sites. The FT-IR spectrum of PEI after adsorption of Cu(II) in the presence of 3.12 mM and 10 mM of tartrates, containing 46.5% and 41.9% of cationic and neutral Cu forms, significantly differs from the PEI-Cu(II) spectrum. Thus, despite relatively low stability constants of Cu(II)-tartrate complex in comparison with PEI-Cu(II) complex, the mechanism of Cu(II) sorption from tartrate solution involves the formation of mixed ligand complexes, whose composition depends on sorption conditions. This fact can limit applicability of the RCD-complex model to a Cu(II) tartrate system, since we hypothesized that one can modify RCD function obtained in water for predictive modeling of sorption in the presence of ligands if the type of Cu(II) binding to the sorption site remains the same. 

### 2.5. Description of PEI Sorption Site Characteristics in Presence of Complexing Agents Using RCD Model 

**Figure 4:**
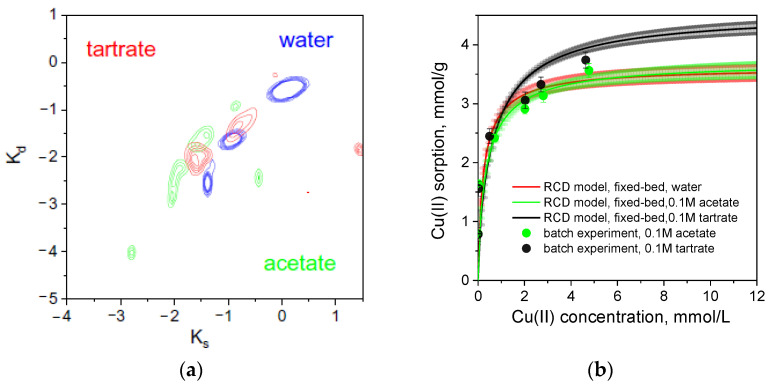
Contour lines with equally spaced contour levels for the distribution of the Cu(II) on PEI cryogel sorption centers for fixed-bed sorption from water, 0.1 M acetate, and 0.1 M tartrate solutions. Here, Ks and Kd represent the logarithms of sorption and desorption rate constants, respectively (**a**). Theoretical isotherms (lines) of Cu(II) sorption on PEI cryogel calculated using Equation (6) from breakthrough curves are shown in Figure 1 (lines) as well as experimental isotherms (dots) obtained in 0.1 M acetate and 0.1 M tartrate solutions in batch at 23 °C, pH = 5.2, solid:liquid ratio of 1:1000, and equilibration time of 72 h (**b**). Color-filled areas in theoretical isotherms show confidence intervals.

First, maps of PEI binding cites distribution in the space of constants of sorption (Ks) and desorption (Kd) rates were calculated using RCD model from breakthrough curves of Cu(II) sorption from water, 0.1 M acetate and 0.1 M tartrate. Under these sorption conditions, RCD function will be calculated for the sorption of [CuL_4_]^6−^ form (>98%) in tartrate solution and mixture of four Cu(II) ionic forms (Table 1) in acetate solution. Site distribution patterns (Figure 4a) with at least two main site types, which differ in sorption rate and affinity, are similar for sorption from all three solutions. This indicates that the same functional fragments were involved in Cu(II) binding. However, one observes a gradual shift of the pattern to the lower sorption rate constants in the water-tartrate-acetate row. Only a small population of the “fast” sorption sites (Ks values > −1) was identified in the acetate solution. Despite an obvious difference in sorption kinetics, Q_max_ values for Cu(II) sorption in 0.1 M acetate and water calculated using Equation (6) of the RCD model (Figure 2b) do not differ significantly and corroborate with maximal dynamic sorption capacities (Table 3). Theoretical isotherms in 0.1 M acetate and tartrate solutions are also in good agreement with experimental data obtained in batch for equilibration time of 72 h (Figure 4b). This shows that all Cu(II) ionic forms in can be adsorbed by PEI but with different rates, and the rate-dependence is more pronounced in 0.1 M acetate solution. 

Participation of the same functional groups in sorption of Cu(II) from solutions with and without complexing agents and the same range of affinity for the sorption centers under these sorption conditions (Figure 4a) allowed for the assumption that RCD function for Cu(II) sorption from water can be used to describe sorption in the presence of complexing agents and predict shape of the breakthrough curves at different flow rates and metal/ligand concentrations, if coefficients a_i,j_. in Equation (13) are found. However, a difference in sorption mechanism of Cu(II) from water and tartrate can negatively affect possibility to calculate one RCD function for the broad range of ligand concentrations, as one can see that the dynamic sorption capacities for Cu(II) significantly depend on the excess of tartrate (Table 3). 

### 2.6. Determination of Sorption Rate Constants for Different Ionic Forms of Cu(II) 

**Figure 5:**
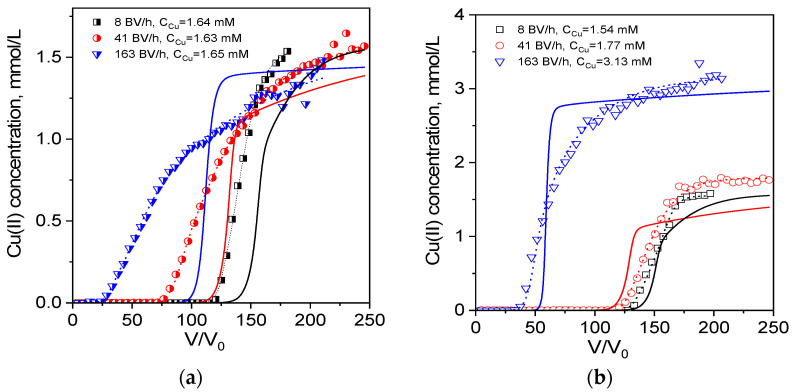
Theoretical breakthrough curves (solid lines) of Cu(II) ion sorption on PEI cryogel from 0.1 M acetate (**a**) and 0.1 M tartrate (**b**) solutions simulated using RCD function for Cu(II) adsorption from water for experimental data presented in Figure 1. Dotted lines (fits of experimental data using the RCD model) are shown for comparison.

Although site distribution maps for Cu(II) sorption in the absence and presence of complexing agents (Figure 4a) do not differ drastically, the direct application of RCD function for Cu(II) adsorption from water for predictive modeling of Cu(II) sorption breakthrough curves in acetate and tartrate solutions does not give satisfactory results (Figure 5). This is especially the case for Cu(II) sorption from acetate solution (Figure 5a) when RCD function for sorption in water cannot predict even the general trend of a breakthrough curve shape and position depending on the flow rate. 

The efficiency of Cu(II) adsorption from 0.1 M acetate, where Cu(II) exists in all four ionic species [CuL_3_]^−1^, [CuL_2_], [CuL]^+^, Cu^2+^ with the lowest contribution of free metal cations (Table 1), shows a strong dependence on the flow rate. This suggests significant differences in sorption rate constants for these various ionic forms. Additional experimental data were obtained for the sorption at Cu(II) excess (0.001 M acetate) and high acetate excess (1 M acetate) to process more breakthrough curves simultaneously using RCD model and obtain one set of a_ij_ coefficients in a Tylor series (Equation (13)), which would fit all breakthrough curves obtained in solutions with different ratios of Cu ionic forms. The Tylor series coefficients calculated for this data set for modifying RCD function for Cu(II) sorption from water are given in Table S1 (Supplementary Materials). It should be mentioned that to shorten computation time, Kd values were limited to −2.5. This is reasonable assumption, since Figure 3a shows that the majority of PEI sorption sites under the chosen sorption conditions fall within this range of desorption rate constants.

**Figure 6:**
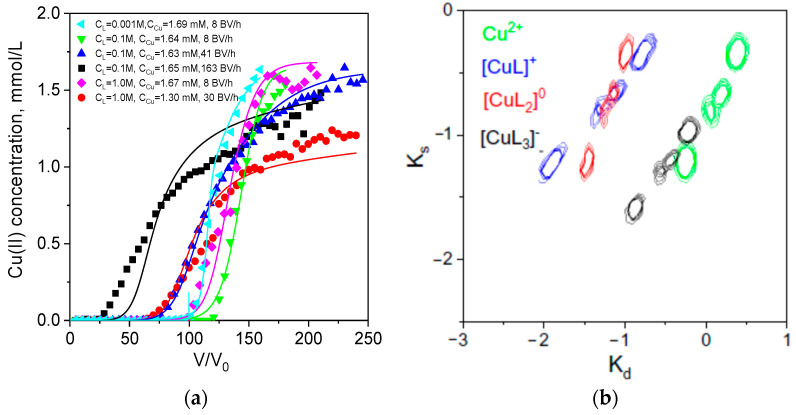
Breakthrough curves of Cu(II) ion sorption on PEI cryogel monoliths in a fixed-bed model from acetate solutions (**a**). Dots are experimental data; lines are fits using the RCD-complex model. Column parameters: diameter is 0.47 cm, bed length is 6 cm, sorbent weight is 0.085 g. Sorption conditions: pH = 5.2, T = 23 °C, flow rates (BV/h) and initial Cu(II) concentrations are as shown in the legend. Distribution of Cu(II)-acetate ionic forms in the space of sorption (Ks)/desorption (Kd) rates constants (**b**).

Figure 6a shows that the RCD-complex model provides a good fit for all breakthrough curves of Cu(II) sorption from acetate solutions varying in ligand concentrations. The corresponding distribution of different Cu(II) ionic forms in the space of sorption/desorption rate constants (Figure 6b) shows that sorption rate constants of the Cu^2+^ ions are notably higher in comparison with those of other ionic forms. The most slowly adsorbed form is a neutral complex [CuL_2_]^0^ that can be attributed to its lack of electrostatic attraction to the amino groups of PEI, which are positively charged at pH 5.2.

**Figure 7:**
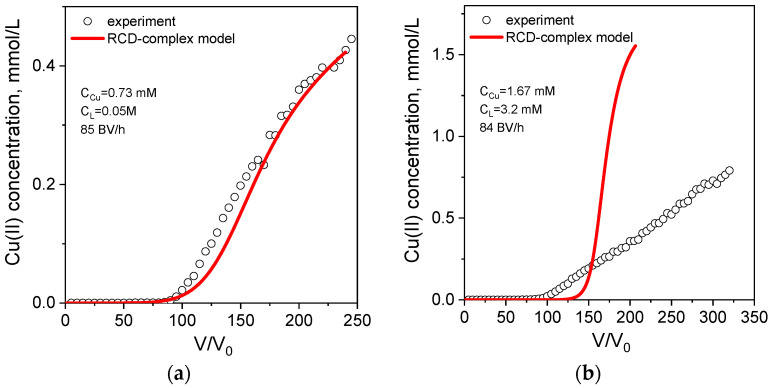
Breakthrough curves (solid lines) of Cu(II) ion sorption on PEI cryogel from acetate (**a**) and tartrate (**b**) solutions simulated using RCD function for Cu(II) adsorption from water. Tylor row coefficients from Tables S1 and S2. Dots are experimental data; sorption conditions are as shown in the legends.

To prove the prognostic value of the RCD-complex model, we have simulated a breakthrough curve of Cu(II) sorption from the solution with Cu(II) and acetate concentrations, which were not used in the experiment designed for the calculation of RCD_complex_ function. The result of predictive breakthrough curve modeling using the RCD function for Cu(II) sorption from water and coefficients of Tylor row is shown in Figure 7a, which demonstrates very good agreement between experimental and theoretical data. 

**Figure 8:**
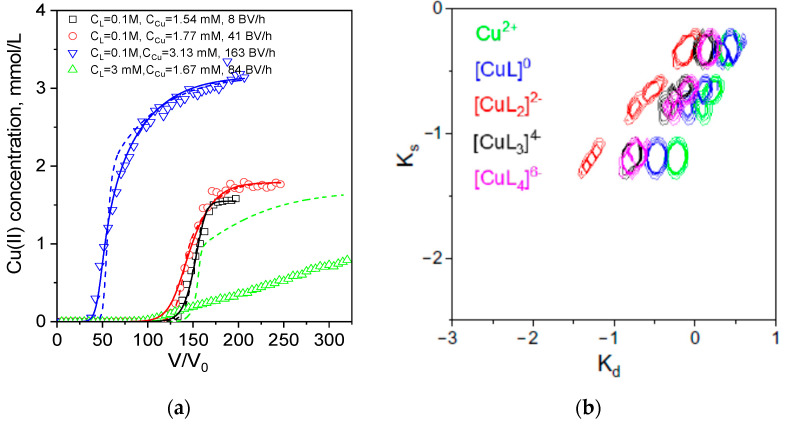
Breakthrough curves of Cu(II) ion sorption on PEI cryogel monoliths in a fixed-bed model from tartrate solutions (**a**). Dots are experimental data; lines are fits using the RCD-complex model with column parameters. Diameter is 0.47 cm, bed length is 6 cm, sorbent weight is 0.085 g. Sorption conditions: pH = 5.2, T = 23 °C, flow rates (BV/h), and initial Cu(II) concentrations were as shown in the legend. Distribution of Cu(II)-tartrate ionic forms in the space of (Ks)/desorption (Kd) rates constants (**b**).

When we extended range of the ligand concentration in tartrate solutions, we failed to find one set of the Tylor row coefficients, which would fit all experimental curves (Figure 8a). At a low tartrate concentration, corresponding to a 2:1 tartrate: Cu(II) mole ratio (Cu speciation is given in Table 2), the breakthrough curve had a very low slope after the breakpoint, while dynamic sorption capacity increased drastically in comparison with sorption from water and 0.1 M tartrate (Table 3). Thus, we have calculated the Tylor row coefficients (Table S2, Supplementary Materials) and distribution of different ion forms in the space of sorption/desorption rate constants for the set of experimental curves obtained in 0.1 M tartrate only (Figure 8b). 

It can be observed that the difference between the sorption rate constant of the free metal ion and other ionic forms, except for [CuL_2_]^2−^, is less pronounced than in Cu(II)-acetate solutions. This observation may indicate a disparity in the Cu(II) sorption mechanism in the presence of these two ligands, which has been demonstrated with FT-IR spectroscopy (Figure 3, Section 2.4). Expectedly, the RCD-complex model with the set of parameters obtained in 0.1 M tartrate failed to predict the shape of the breakthrough curve at low tartrate concentration (Figure 7b). 

At this point, it is important to emphasize that assumptions made in the RCD-complex model, first of all, are based on the fact that despite the difference in ionic speciation of the metal ion, the adsorbate binds to the sorbent via the same mechanisms in water and in the presence of a complexing agent. In other words, sorption of metal ion and metal-chelate results in binding of a metal ion by the same functional groups of the sorbent and the release of the ligand in the latter case. Most likely, this necessary condition is not fulfilled in a broad range of tartrate concentrations. In such cases, the necessary conditions for application of the RCD-complex model are not fulfilled, making it impossible to find a single set of parameters for predictive modeling of sorption kinetics.

Thus, the decision on applicability of RCD-complex model for predictive modelling of sorption kinetics in a broad range of solution compositions and sorption conditions should be based on information about the sorption mechanism. If all ionic forms of metal ion form with the sorption center complexes of the same or a very similar configuration, the RCD-complex model can be used for predictive modeling of sorption dynamics. 

## 3. Materials and Methods

### 3.1. Materials

Branched polyethylenimine (PEI) with the average molecular weight of 25 kDa and cross-linking reagents—ethylene glycol diglycidyl ether (DGE-EG) and 1,4-butanediol diglycidyl ether (DGE-1,4-BD)—were purchased from Sigma–Aldrich (St. Louis, MO, USA). Other reagents—HCl, NaOH, metal salts—were of an analytical grade. 

### 3.2. Cryogel Synthesis and Characterization

The PEI cryogels were obtained as described in detail in [35]. Briefly, the solution for cryogel fabrication was prepared by addition of the cross-linker at the DGE-1,4-BD:PEI molar ratio 1:4 to the 5% PEI solution under intensive stirring at room temperature. The plastic syringes of diameters (0.47 cm) were filled with the mixed solution and kept frozen at −20 °C for 7 days. After thawing at room temperature, the obtained PEI cryogels were washed with 10 mL of 0.1 M NaOH solution and distilled water using peristaltic pump (Ismatec, Wertheim, Germany). 

Fourier transform infrared (FT-IR) spectra for air-dried PEI cryogel before and after Cu(II) sorption were recorded using an IR Affinity-1 spectrometer with QATR 10 single reflection ATR accessory (Shimadzu, Kyoto, Japan). The monolith cryogels after the Cu(II) sorption were thoroughly washed with a large volume of distilled water to remove all unbound ions. 

### 3.3. Investigation of the Sorption in Fixed-Bed and Sorption Isotherms 

The dynamics of Cu(II) ion sorption on monolith PEI cryogel was investigated by pumping solution of copper nitrate in water, sodium acetate, or potassium–sodium tartrate solutions through the sorption column at different flow rates. Prior to solution feeding, the column was equilibrated by passing 10 mL of the background solution without Cu(II) added. The samples were collected every 5 mL, and the copper concentrations were determined by atomic absorption spectrometry (AAS) using a AA-6200 Atomic Absorption Flame Emission Spectrophotometer (Shimadzu, Kyoto, Japan) device.

The sorption isotherms were investigated at solid:liquid ratio 1:1000, the contact time was 72 h, and the solutions with sorbents were agitated using a Biosan PSU-20i orbital shaker (Riga, Latvia) at 200 rpm. At least three replicates were made to ensure the results reproducibility. The adsorbed amounts were calculated using the difference in initial and equilibrium concentrations of the metal ions in the solutions determined by AAS. 

### 3.4. Data Analysis 

#### 3.4.1. Calculation of RCD Functions for Metal Ions in the Presence of Complexing Agents

To obtain the RCD function from sorption dynamics experiments, several breakthrough curves obtained at different flow rates and fixed ligand concentration (0.1 M) were processed using the same RCD model, which was used to describe Cu(II) sorption in a fixed-bed application in the absence of complexing agent [29]. Using simulation modeling as described in [29], an averaged 3D distribution functions for Cu(II) sorption sites of PEI in presence of complexing agents (acetate and tartrate) were plotted as contour lines in the space of sorption/desorption rates constants—ρ(pK_s, p_K_d_),pK_s_, pK_d_. 

Alternatively, experimental breakthrough curves obtained at different ligands concentrations and flow rates were processed using RCD function obtained earlier for Cu(II) sorption from aqueous solution [29], and Equation (15) to calculate the coefficients $a_{ij}$ for every complex form. On figures describing the distribution of sorption centers of different types in the pK_s_-pK_d_ space, the deter position coincides with the circle center. Here, the circle radius is proportional to the maximal capacity of this very center ($q^{\max}$). 

#### 3.4.2. Predictive Modeling Using RCD Model 

The predictive modeling of the breakthrough curves for different initial concentrations of the adsorbate and ligands have been performed using the RCD function determined from fixed-bed experiments for Cu(II) from water or using RCD_complex_ function calculated from fixed-bed experimental data for solutions at different ligand concentrations and flow rates. 

## 4. Conclusions

Aside from affinity, selectivity, and high-sorption capacity, the kinetic characteristics of sorbents are of utmost importance, as they determine productivity of both industrial water treatment facilities and small-size, point-of-use (POU) filters operated at high flow rates. In this work, we have discussed how and when one can predict breakthrough curves of metal ion sorption in the presence of complexing agents, if diffusion limitations are eliminated via well-developed porous structure of the sorbent, and if chemisorption is a rate-limiting stage. 

A new version of the earlier developed and verified extended Rate Constants Distribution (RCD) model [27,28,29], which additionally includes complex-formation equilibria, was discussed here and applied to an investigation of Cu(II) sorption kinetics on a supermacroporous monolith polyethyleneimine (PEI) cryogel in the presence of acetate and tartrate. The RCD-complex model assumes that in order to predict the shape of the breakthrough curves in the presence of a ligand, one can use the parameters (RCD-function) determined for the metal ion sorption from ligand-free solutions along with a set of coefficients, which account for the variation in sorption rate constants of different metal ionic forms. However, this approach assumes the same or a very similar configuration of the metal–sorbent site after adsorption from solution with and without a complexing agent. Thus, information on sorption mechanism is crucial for the decision on applicability of the model. 

The application of the RCD-complex model to the sets of Cu(II) sorption breakthrough curves obtained at different flow rates and metal speciation in tartrate and acetate solutions allowed us to conclude the following points: (i) despite the lower stability of Cu(II)-acetate complex, it can have a more pronounced negative effect on sorption kinetics than tartrate at high ligand:metal ratios; (ii) the neutral Cu(II)-acetate complex adsorbs with the lowest rate; (iii) the presence of tartrate results in a twofold increase in the dynamic sorption capacity of PEI for Cu(II) at low ligand:metal ratios even at high flow rates (up to 130 BV/h); and (iv) the RCD-complex model successfully predicts breakthrough curves of Cu(II) sorption in the broad concentration range of acetate but not of tartrate. The latter fact was related to alteration of sorption mechanism in the presence of tartrate and formation of the mixed ligand Cu(II)-PEI-tartrate complexes instead of Cu(II)-PEI complex, despite a large difference between formation constants of Cu(II)-PEI and Cu(II)-tartrate complexes. 

## Figures and Tables

**Table 1:** Cu(II) speciation in acetate solutions, pH = 5.2. Calculations performed using Phreeqc Interactive 3.7.3-15968 software *.

		Cu, 1.56 mM, L, 0.001 M	Cu, 1.56 mM, L, 0.1 M	Cu, 1.56 mM, L, 1.0 M	Cu, 0.78 mM, L, 0.05 M
L = Acetate	Cu ^2+^ [CuL]^+^	91.0% 8.27%	3.90% 22.7%	0.03% 0.866%	11.2% 39.1%
	[CuL_2_]^0^	0.13%	29.1%	6.95%	28.1%
	[CuL_3_]^−1^	0.0017%	44.3%	92.2%	21.5%

* Cumulative complex stability constants logarithms: K_1_ = 2.27, K_2_ = 3.73, K_3_ = 5.05 [44].

**Table 2:** Cu(II) speciation in tartrate solutions, pH = 5.2. Calculations performed using Phreeqc Interactive 3.7.3-15968 software *.

		Cu, 1.56 mM, L, 0.1 M	Cu, 3.12 mM, L, 0.1 M	Cu, 1.56 mM, L, 0.01 M	Cu, 1.56 mM, L, 3.12 mM
L = Tartrate	Cu^2+^	0.0047%	0.00583%	17.9%	46.5%
	[CuL]	0.025%	0.02%	38.6%	41.9%
	[CuL_2_]^2−^	0.233%	0.239%	37.5%	11.1%
	[CuL_3_]^4−^	1.43%	1.42%	4.62%	0.236%
	[CuL_4_]^6−^	98.3%	98.3%	1.28%	0.007%

* Cumulative complex stability constants logarithms: K_1_ = 3.0, K_2_ = 5.11, K_3_ = 5.76, K_3_ = 6.20 [44].

**Table 3:** Dynamic sorption capacities for Cu(II) ions on monolith PEI cryogel (experimental data are shown in Figure 1 and Figure 2).

	C_Cu_, mmol/L	C_L_, mol/L	Flow Rate, BV/h	Q_eff_, mmol/g *	Q_max_, mmol/g *
Water	3.15	0	17(8)	2.43	2.95
	1.56	0	41	1.78	2.42
	1.56	0	163	1.59	1.87
L = Acetate	1.64	0.1	8	2.32	2.90
	1.63	0.1	41	1.42	2.64
	1.65	0.1	163	0.48	2.16
	1.69	0.001	8	1.99	2.58
	1.67	1	8	1.90	2.77
	1.3	1	30	1.03	2.09
L = Tartrate	1.54	0.1	8	2.40	2.84
	1.77	0.1	41	2.38	2.90
	3.13	0.1	163	1.25	2.85
	1.57	0.01	130	3.87	5.61
	1.65	0.00312	8	>5.41	>5.41
	1.67	0.00312	84	1.73	>5.44

* Q_eff_—effective dynamic sorption capacity for the breakthrough point of 2 mgCu/L; Q_max_—maximal dynamic sorption capacity.

## Data Availability

Data are available from the authors upon request.

## Supplementary Materials

The following supporting information can be downloaded at: https://www.mdpi.com/article/10.3390/ijms241512385/s1.

## Appendix A

Expansion in a Taylor series is conducted around the point $\left(pKs_{Me}^{0},pKd_{Me}^{0}\right)$:

(A1) $$\begin{array}{l} pKs_{Me}^{0}=\sum_{i=0}^{N_{b}-1}r_{i}\cdot pKs_{Me,i}\\ pKd_{Me}^{0}=\sum_{i=0}^{N_{b}-1}r_{i}\cdot pKd_{Me,i} \end{array}$$

$N_{b}—$the number of basis centers; $pKs_{Me,i},pKd_{Me,i}—$the coordinates of the basis center i of the RCD function of sorption of metal ions. $r_{i}—$the RCD function value in the point $\left(pKs_{Me,i},pKd_{Me,i}\right)$. Let us define the function of transformation of logarithms of the sorption rate constants as:

(A2) $$\begin{array}{l} pKs_{MeL_{n}}=F_{n}\left(pKs_{Me},pKd_{Me}\right)\\ pKs_{Me}=F_{0}\left(pKs_{Me},pKd_{Me}\right) \end{array}$$

Then, the expansion of the function of transformation of the logarithm of the rate constant for the sorption of the complex form in a Taylor series can be written as:

(A3) $$\begin{array}{l} pKs_{MeL_{n}}=F_{n}\left(pKs_{Me}^{0},pKd_{Me}^{0}\right)+\frac{\partial F_{n}\left(pKs_{Me}^{0},pKd_{Me}^{0}\right)}{\partial pKs_{Me}}\left(pKs_{Me}-pKs_{Me}^{0}\right)+\\ \frac{\partial F_{n}\left(pKs_{Me}^{0},pKd_{Me}^{0}\right)}{\partial pKd_{Me}}\left(pKd_{Me}-pKd_{Me}^{0}\right)+\frac{1}{2}\frac{\partial^{2}F_{n}\left(pKs_{Me}^{0},pKd_{Me}^{0}\right)}{\partial pKs_{Me}{}^{2}}{\left(pKs_{Me}-pKs_{Me}^{0}\right)}^{2}+\ldots \end{array}$$

After removal of brackets and grouping of terms by equal powers in (A3), one obtains:

(A4) $$\begin{array}{l} pKs_{MeL_{n}}=a_{00}^{n}+a_{10}^{n}pKs_{Me}+a_{01}^{n}pKd_{Me}+a_{20}^{n}pKs_{Me}{}^{2}+a_{11}^{n}pKs_{Me}pKd_{Me}+\ldots \\ a_{00}^{n}=F_{n}\left(pKs_{Me}^{0},pKd_{Me}^{0}\right)-\frac{\partial F_{n}\left(pKs_{Me}^{0},pKd_{Me}^{0}\right)}{\partial pKs_{Me}}pKs_{Me}^{0}-\frac{\partial F_{n}\left(pKs_{Me}^{0},pKd_{Me}^{0}\right)}{\partial pKd_{Me}}pKd_{Me}^{0}+\ldots \\ a_{10}^{n}=\frac{\partial F_{n}\left(pKs_{Me}^{0},pKd_{Me}^{0}\right)}{\partial pKs_{Me}}\\ a_{01}^{n}=\frac{\partial F_{n}\left(pKs_{Me}^{0},pKd_{Me}^{0}\right)}{\partial pKd_{Me}}\\ \ldots \end{array}$$
