# Supplementary material for: Extended Rate Constants Distribution (RCD) Model for Sorption in Heterogeneous Systems: 4. Kinetics of Metal Ions Sorption in the Presence of Complexing Agents—Application to Cu(II) Sorption on Polyethyleneimine Cryogel from Acetate and Tartrate Solutions
Source: Int J Mol Sci. 2023 Aug 3;24(15):12385. doi: 10.3390/ijms241512385 (PMC10418622; doi:10.3390/ijms241512385)
Supplement: Supplementary file 1 [file ijms-24-12385-s001.zip › ijms-2508876-supplementary.pdf]

Supplementary materials

Extended Rate Constants Distribution (RCD) Model for Sorption in Heterogeneous Systems: 4. Kinetics of Metal Ions Sorption in the Presence of Complexing Agents. Application to Cu(II) Sorption on Polyethyleneimine Cryogel from Acetate and Tartrate Solutions

Alexey Golikov, Yuliya Privar, Denis Balatskiy, Natalia Polyakova, and Svetlana Bratskaya *

Institute of Chemistry, Far Eastern Branch of Russian Academy of Sciences,
159, prospect 100-letiya Vladivostoka, 690022 Vladivostok, Russia;
glk@ich.dvo.ru (A.G.)

* Correspondence: sbratska@ich.dvo.ru

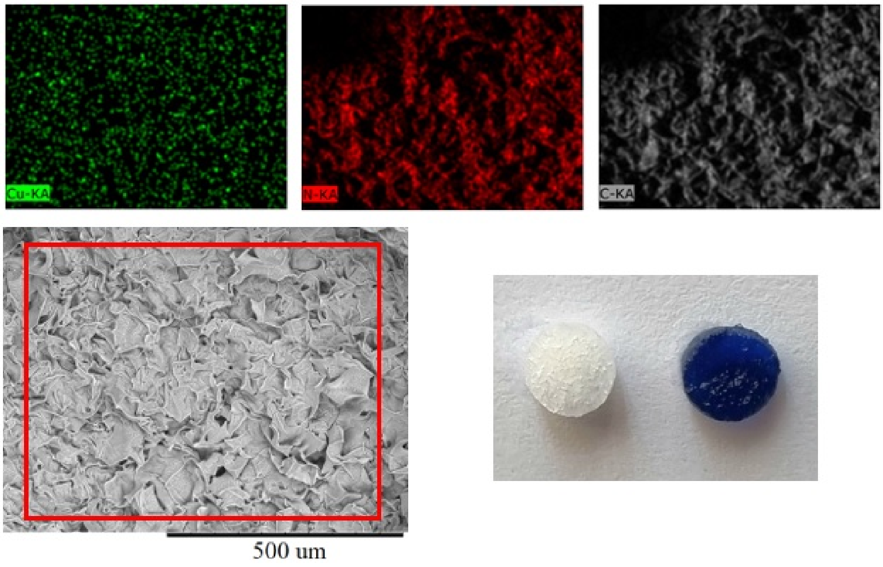

(a)

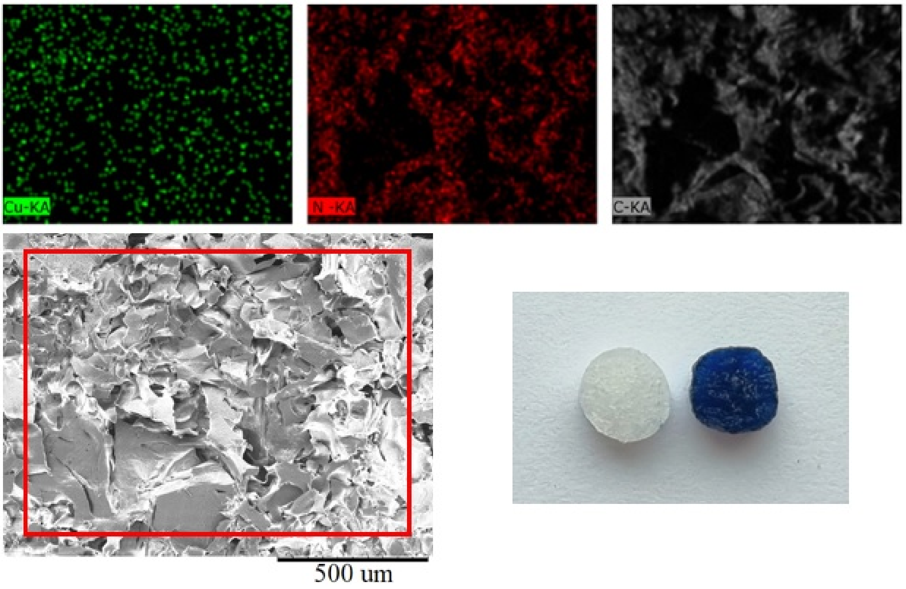

(b)

Figure S1. SEM images, EDX maps and photos before and after Cu(II) sorption from 0.1M acetate(a) and 0.1M tartrate (b) solutions.

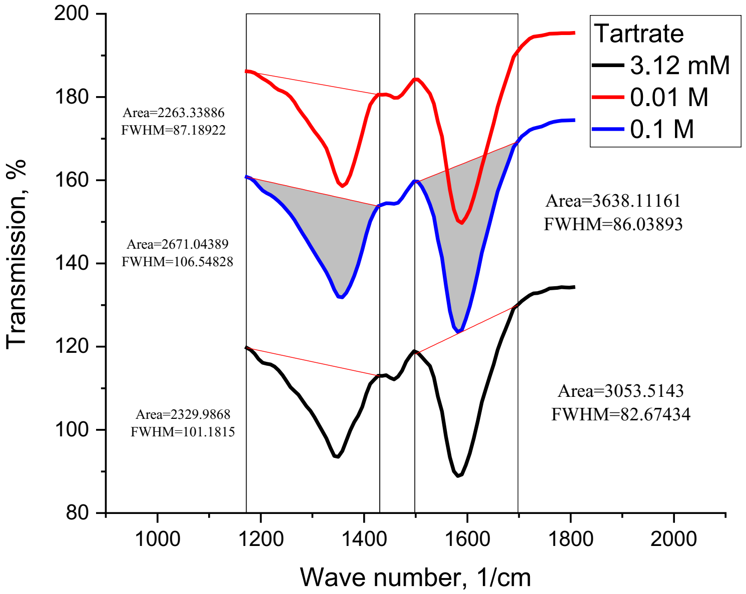

Figure S2. Peak widths and areas for FT-IR spectra of PEI cryogels after Cu(II) sorption from 0.1M tartrate at 41 BV/h, 0.01M tartrate at 130 BV/h, 3.12 mM tartrate at 8 BV/h.

Table S1. Tylor series coefficients for description of Cu(II)-acetate sorption on PEI using basis RCD function for Cu(II) sorption from water

	a_{00}	a_{10}	a_{01}	a_{20}	a_{11}	a_{02}	a_{30}	a_{21}	a_{12}	a_{03}
MeL	-0.396449	1.01422	0.236004	0.0633601	-0.0141715	-0.171757	-0.114591	-0.126626	0.0146045	0.0659919
MeL_2	-0.337711	0.888904	0.391048	-0.0941893	0.0867348	-0.193622	-0.0500305	-0.140627	-0.110772	0.0997239
MeL_3	-0.299943	0.966508	0.293557	0.0114103	-0.0703197	-0.0402011	-0.070993	-0.0396597	-0.0181736	0.00827892

Table S2. Tylor series coefficients for description of Cu(II)-tartrate sorption on PEI using basis RCD function for Cu(II) sorption from water

	a_{00}	a_{10}	a_{01}	a_{20}	a_{11}	a_{02}
MeL	-0.063	1.14247	0.109306	0.390001	-0.0218159	0.0152271
MeL_2	-0.35821	0.996755	0.251395	0.286133	0.315041	-0.298722
MeL_3	-0.274294	1.00252	0.0908478	0.285833	-0.0150183	-0.104897
MeL_4	-0.185573	1.04245	0.00929065	-0.132211	-0.0137171	-0.161819

Table S3. Cu(II) speciation in tartrate solutions (assuming presence of bi-nuclear complexes), pH=5.2. Calculations performed using Phreeqc Interactive 3.7.3-15968 software*

		Cu 100 mg/l, Tartrate 0.1 M	Cu 200 mg/l, Tartrate 0.1 M	Cu 100 mg/l, Tartrate 0.00312 M
L=Tartrate	Cu^{2+}	4.71e-3%	5.83e-3%	46.5%
	[CuL]	2.46e-2%	2.72e-2%	41.9%
	$[CuL_2]^{2-}$	0.233%	0.239%	11.1%
	$[CuL_3]^{4-}$	1.43%	1.42%	0.236%
	$[CuL_4]^{6-}$	98.3%	98.3%	7.20e-3%
	$[Cu_2L_2]^0$	3.68e-6%	9.06e-6%	7.45%
	$[Cu_2L_3]^{2-}$	2.53e-6%	5.76e-6%	0.138%
	$[Cu_2L_4]^{4-}$	2.05e-6%	4.51e-4%	3.69e-2%

* Cumulative complex stability constants logarithms for bi-nuclear complexes were taken from [1]

References

1. Bottari, E.; Liberti, A.; Rufolo, A. On the Formation of CuII-Tartrate Complexes in Acid Solution. *Inorganica Chim. Acta* **1969**, *3*, 201–206, doi:10.1016/s0020-1693(00)92480-0.
